# Impacts of hemoperfusion combined with continuous renal replacement therapy on renal function and immune function in patients with acute renal failure caused by poisoning

**DOI:** 10.3389/fmed.2026.1827840

**Published:** 2026-05-20

**Authors:** Hui Fang, Jingzhi Huang, Shufang Fan, Yanlin Xu, Juanjun Liu

**Affiliations:** 1 Department of Nephrology, The First People's Hospital of Jiande, Jiande, Zhejiang, China; 2 School of Data Science and Artificial Intelligence, Wenzhou University of Technology, Wenzhou, Zhejiang, China

**Keywords:** acute renal failure, continuous renal replacement therapy, hemoperfusion, immune function, poisoning, renal function

## Abstract

**Aim:**

To assess the clinical efficacy of hemoperfusion (HP) plus continuous renal replacement therapy (CRRT) in patients with poisoning-induced acute renal failure (ARF), with particular focus on renal function recovery and immune function modulation.

**Methods:**

One hundred and sixteen patients with poisoning-induced ARF admitted to our hospital from March 2022 to March 2025 were enrolled and randomly divided into the single group receiving CRRT alone and the study group receiving HP + CRRT. The primary outcome was the reduction in serum creatinine (Scr) at 72 h post-treatment. Secondary outcomes included blood urea nitrogen (BUN), 24-h urine output, time to CRRT independence, proportion of patients achieving complete renal recovery at 28 days, immune function parameters, inflammatory markers, disease severity scores, clinical outcomes, and adverse events.

**Results:**

The combination group presented better renal function recovery, with lower serum creatinine and blood urea nitrogen levels at 48 h, 72 h, and 7 days post-treatment, and higher 24-h urine output at all time points (*p* < 0.05). Time to CRRT independence was significantly shorter in the combination group (*p* < 0.001), and the percentage of patients achieving complete renal recovery at 28 days was significantly higher (82.8% vs. 62.1%, *p* = 0.014). At 7 days post-treatment, the combination group demonstrated significantly improved immune function, with higher CD3^+^, CD4^+^, CD4^+^/CD8^+^ ratio, and immunoglobulin levels (IgG, IgA, IgM) (*p* < 0.05 for all), and lower CD8^+^ (*p* < 0.05). Inflammatory markers (CRP, PCT, IL-6) were significantly lower in the combination group at 48 and 72 h (*p* < 0.05). The combination group also had lower APACHE II and SOFA scores at 7 days (*p* < 0.001), shorter ICU stay (*p* < 0.001), shorter hospital stay (*p* < 0.001), lower 28-day mortality (10.3% vs. 24.1%, *p* = 0.048), and fewer adverse events (17.2% vs. 32.8%, *p* = 0.016).

**Conclusion:**

HP combined with CRRT significantly improves renal function recovery and enhances immune function in patients with poisoning-induced ARF compared to CRRT alone, potentially by removing circulating toxins and inflammatory mediators while modulating the systemic inflammatory response.

## Introduction

Acute renal failure (ARF), also referred to as acute kidney injury (AKI), belongs to a critical clinical syndrome featured by a rapid decrease in glomerular filtration rate, resulting in the accumulation of metabolic waste products, electrolyte imbalances, as well as fluid homeostasis disruption (1). Among its various etiologies, poisoning-induced ARF represents a particularly severe and life-threatening condition, accounting for a substantial proportion of intensive care unit admissions worldwide (2). Common nephrotoxic agents include pesticides, heavy metals, industrial chemicals, and pharmaceutical overdoses, which can directly damage renal tubular epithelial cells, induce vasoconstriction, and trigger inflammatory cascades that exacerbate kidney injury (3). The mortality rate associated with poisoning-induced ARF remains alarmingly high, particularly in cases where timely and effective renal replacement therapy is not promptly initiated (4).

In addition to its direct effects on renal function, severe poisoning frequently induces systemic inflammatory responses and immune dysfunction, which further complicate clinical management and adversely affect patient prognosis (5). The dysregulation of immune function in these patients is characterized by alterations in both cellular and humoral immunity, including imbalances in T lymphocyte subsets, impaired phagocytic activity, and abnormal cytokine profiles (6). These immunological disturbances not only increase susceptibility to secondary infections but also contribute to the propagation of multi-organ dysfunction syndrome, creating a vicious cycle that perpetuates renal injury and systemic deterioration (7).

Recently, continuous renal replacement therapy (CRRT) has become a cornerstone in treating critically ill patients with ARF, offering the advantages of gradual solute removal, hemodynamic stability, and continuous acid–base and electrolyte correction (8). CRRT effectively clears small molecular weight toxins and helps maintain internal milieu homeostasis, yet its efficacy in removing protein-bound or larger molecular weight toxins commonly encountered in severe poisoning cases may be limited (9). Hemoperfusion (HP), alternatively known as hemoadsorption, utilizes adsorbent materials such as activated charcoal or synthetic resins to directly remove circulating toxins, including those with high protein-binding affinity (10). By integrating HP + CRRT, it is theoretically possible to achieve synergistic benefits: enhanced toxin clearance through adsorption combined with sustained metabolic and volume control through continuous dialysis (11).

Emerging evidence suggests that this combined therapeutic approach may not only improve renal recovery rates but also modulate the systemic inflammatory response and preserve immune function in critically ill patients (12). However, the specific impact of HP combined with CRRT on both renal and immune parameters in patients with poisoning-induced ARF remains inadequately characterized.

Consequently, the present research aims to assess the clinical efficacy of HP combined with CRRT in patients with ARF secondary to poisoning, with particular focus on its effects on renal function recovery and immune system modulation.

## Methods

### Study design

This prospective randomized controlled study was implemented from March 2022 to March 2025. The study protocol was approved by the Ethics Committee of our hospital. Written informed consent was obtained from all participants or their families before enrollment.

Inclusion criteria: (1) Patients aged 18–75 years; (2) Confirmed history of acute poisoning (pesticides, pharmaceuticals, or other nephrotoxic agents) with detectable plasma toxin levels; (3) Diagnosis of acute renal failure according to KDIGO criteria (increase in serum creatinine ≥0.3 mg/dL within 48 h or ≥1.5 times baseline within 7 days, or urine output <0.5 mL/kg/h for 6 h); (4) Admission within 24 h of poisoning; (5) Requirement for CRRT as determined by the attending physician.

Exclusion criteria: (1) Pre-existing chronic kidney disease (estimated glomerular filtration rate <60 mL/min/1.73m^2^ for >3 months); (2) End-stage renal disease requiring maintenance dialysis; (3) Severe pre-existing immune deficiency disorders or long-term immunosuppressant use; (4) Malignancy; (5) Pregnancy or lactation; (6) Contraindications to anticoagulation; (7) Expected survival <24 h.

### Sample size calculation

Sample size was calculated based on the primary outcome of serum creatinine reduction at 72 h post-treatment (13). With *α* = 0.05 (two-tailed) and power (1-β) = 0.80, the estimated required sample size was 49 patients per group. Accounting for a 15% dropout rate, a total of 116 patients (58 per group) were included.

### Randomization and group allocation

Eligible patients were randomly divided into the single group (CRRT alone, *n* = 58) or the combination group (HP + CRRT, *n* = 58) utilizing a computer-generated random number table. Allocation concealment was ensured by means of sequentially numbered, opaque, sealed envelopes.

### Rationale for the CRRT-only control group

In this study, we selected CRRT alone as the control intervention because not all poisoning agents in our cohort (e.g., small water-soluble molecules such as methanol or lithium) are effectively removed by hemoperfusion, and current clinical guidelines do not mandate HP for every case of poisoning-induced acute renal failure. The CRRT-only group allowed us to isolate the incremental therapeutic effect of adding HP to standard continuous renal replacement therapy. All enrolled patients met the criteria for CRRT initiation, and the decision to withhold HP in the control group was determined solely by randomization, not by clinical judgment of futility. This design provides a rigorous assessment of the additive efficacy of HP + CRRT, although we acknowledge that in centers where HP is considered standard of care, the control intervention may be viewed as less than optimal.

### Interventions

Single group (CRRT alone): Patients adopted continuous veno-venous hemodiafiltration (CVVHDF) using a Prismaflex system (Gambro, Sweden) with an AN69 filter. Vascular access was obtained via a double-lumen central venous catheter inserted into the internal jugular or femoral vein. The treatment parameters were as follows: blood flow rate 150–200 mL/min, dialysate flow rate 1,000–1,500 mL/h, replacement fluid rate 1,000–1,500 mL/h (pre-dilution), and net ultrafiltration rate according to clinical requirements. Regional citrate anticoagulation or low molecular weight heparin was used based on patient bleeding risk. CRRT was continued until renal function recovery or for a maximum of 14 days.

Study group (HP + CRRT): Patients received hemoperfusion in series with CRRT. A hemoperfusion cartridge containing neutral macroporous resin adsorbent (HA330, Jafron Biomedical, China) was placed proximal to the dialyzer in the CRRT circuit. HP was performed for 2–3 h daily for the first 3 days of treatment, after which the cartridge was removed and CRRT continued alone. The remaining CRRT parameters were identical to the single group. All protocol-specified blood samples at 24, 48, and 72 h were collected at 6:00 a.m., prior to the start of the daily hemoperfusion session and approximately 20 h after the completion of the previous hemoperfusion, thereby reflecting a steady-state inter-treatment interval.

### Anticoagulation protocol

Anticoagulation was selected based on bleeding risk and liver function. Regional citrate anticoagulation (RCA) was preferred; low molecular weight heparin (LMWH) was used when RCA was contraindicated. For RCA, 4% trisodium citrate was infused pre-filter at 1.5–2.0 mmol/L blood flow, targeting post-filter ionized calcium (iCa) of 0.25–0.35 mmol/L and systemic iCa of 0.9–1.1 mmol/L via calcium infusion. iCa was monitored at 2 h after initiation and then every 6 h. In patients with liver dysfunction (Child-Pugh B/C or bilirubin >51 μmol/L) or high lactate (>4 mmol/L), total Ca/iCa ratio was monitored every 6 h; a ratio >2.5 indicated citrate accumulation and prompted switching to LMWH. For hypotensive patients (MAP <60 mmHg despite vasopressors), LMWH was preferred. For LMWH (nadroparin), an initial bolus of 15–20 anti-Xa IU/kg was given intravenously, followed by continuous infusion at 5–10 anti-Xa IU/kg/h, targeting anti-Xa activity of 0.25–0.35 IU/mL for patients with normal bleeding risk and 0.20–0.30 IU/mL for those with increased bleeding risk. Anti-Xa activity was measured 4 h after the bolus and then once daily. For patients with severe liver dysfunction or platelets <30 × 10^9^/L, the target was reduced to 0.15–0.25 IU/mL. Safety thresholds and stopping rules for HP were as follows: circuit clotting before completing 2 h led to session termination, and two consecutive clotting events within 24 h prompted a change of anticoagulant; major bleeding (intracranial, gastrointestinal requiring transfusion, or hemoglobin drop >2 g/dL within 24 h) led to immediate discontinuation of anticoagulation and HP, while minor bleeding led to reduced anticoagulation targets; hypotension with MAP <55 mmHg despite fluid resuscitation and vasopressors resulted in HP termination (CRRT alone could continue); a platelet drop >50% from baseline or to <20 × 10^9^/L triggered cessation of HP and LMWH with evaluation for heparin-induced thrombocytopenia; technical failure such as cartridge saturation or pressure >150 mmHg led to early HP termination with continuation of CRRT alone.

### Nursing management

A dedicated team of intensive care unit nurses with specialized training in CRRT and HP provided comprehensive nursing care throughout the treatment period. Pre-treatment nursing included assessment of vital signs, fluid status, and vascular access, as well as patient and family education about the procedures and potential complications. During treatment, vital signs (heart rate, blood pressure, respiratory rate, oxygen saturation) and the CRRT circuit (for clotting, air bubbles, or disconnections) were continuously monitored; for patients in the combination group, the hemoperfusion cartridge was also observed for saturation and pressure changes. Fluid balance was recorded every 2 h. Anticoagulation status was assessed regularly (ACT or APTT) and adjusted according to the protocol. Strict aseptic technique was maintained for central venous catheter care, with daily inspection of the insertion site. Complications such as bleeding, hypotension, or suspected catheter-related bloodstream infection were managed according to established protocols. Nutritional status was assessed daily with enteral or parenteral nutrition provided as needed, and psychological support and family communication were offered. After each session, patients were assessed for hemodynamic stability, bleeding signs, and catheter site condition before circuit discontinuation. All assessments and interventions were documented in the electronic medical record system. Detailed nursing procedures are provided in Supplementary file 1.

### Outcome measures

#### Primary outcome

The primary outcome was the reduction in serum creatinine (Scr) at 72 h post-treatment, which was used for sample size calculation.

#### Secondary outcomes

##### Renal function parameters

Blood urea nitrogen (BUN) and 24-h urine output measured at baseline, 24, 48, and 72 h, as well as 7 days post-treatment; time to CRRT independence; proportion of patients with complete renal recovery (return to baseline Scr) at 28 days.

##### Immune function parameters

T lymphocyte subsets (CD3^+^, CD4^+^, CD8^+^ percentages and absolute counts, CD4^+^/CD8^+^ ratio) measured by flow cytometry at baseline and 7 days post-treatment; serum immunoglobulins (IgG, IgA, IgM) measured by immunoturbidimetry.

##### Inflammatory markers

C-reactive protein (CRP), procalcitonin (PCT), interleukin-6 (IL-6) detected at baseline, 24, 48, and 72 h post-treatment.

##### Disease severity scores

Acute Physiology and Chronic Health Evaluation II (APACHE II) and Sequential Organ Failure Assessment (SOFA) scores calculated at baseline and 7 days post-treatment.

##### Clinical outcomes

Duration of CRRT, intensive care unit (ICU) stay, hospital stay, 28-day mortality, as well as adverse events.

### Statistical analysis

Statistical analyses were performed using SPSS software (version 26.0). Continuous variables were tested for normality using the Shapiro–Wilk test and are presented as mean ± standard deviation (SD) for normally distributed data; non-normally distributed variables were analyzed after appropriate transformation or using non-parametric tests as indicated. Categorical variables are presented as frequencies and percentages. For between-group comparisons, independent-sample t-tests were used for normally distributed continuous variables, and the Mann–Whitney U test was used for non-normally distributed variables. Chi-square tests or Fisher‘s exact tests were used for categorical variables. For repeated-measures data (renal function parameters and inflammatory markers measured at baseline, 24, 48, 72 h, and day 7), a two-way repeated-measures analysis of variance (ANOVA) was employed with time as the within-subjects factor and group as the between-subjects factor. When the time × group interaction was significant, post-hoc pairwise comparisons were performed using Bonferroni correction. To account for multiple comparisons across the three primary time points of interest (48 h, 72 h, and day 7) for renal outcomes, the significance threshold was adjusted to *α* = 0.017 (0.05/3). For secondary outcomes (immune parameters, inflammatory markers at individual time points, disease severity scores, and clinical outcomes), we applied Bonferroni correction for multiple comparisons where appropriate. Specifically, for the 8 immune parameters (CD3^+^, CD4^+^, CD8^+^, CD4^+^/CD8^+^, IgG, IgA, IgM, and the three inflammatory markers at each time point), a corrected threshold of *p* < 0.00625 (0.05/8) was used for the primary comparisons between groups at day 7. All statistically significant findings reported in the Results remained significant after this correction, except where explicitly noted as non-significant. A *p*-value < 0.05 was considered statistically significant for primary outcomes and for comparisons that did not require correction; for corrected comparisons, the adjusted thresholds are indicated above. All tests were two-tailed.

## Results

### Participant flow and baseline characteristic

A total of 142 patients with poisoning-induced acute renal failure were screened for eligibility. Of these, 26 patients were excluded: 14 did not meet the inclusion criteria (10 had pre-existing chronic kidney disease, 4 had expected survival <24 h), 8 declined to participate, and 4 had contraindications to anticoagulation. The remaining 116 patients were randomly assigned to the single group (CRRT alone, *n* = 58) or the combination group (HP + CRRT, *n* = 58). No patient was lost to follow-up during the 28-day study period. All 116 patients were included in the final analysis on an intention-to-treat basis. Supplementary Figure 1 shows the CONSORT flow diagram of participant enrollment, randomization, and follow-up. Baseline characteristics were comparable between the two groups, with no significant differences (*p* > 0.05), indicating successful randomization (Table 1).

**Table 1:** Baseline characteristics of patients.

Characteristic	Single group (*n* = 58)	Combination group (*n* = 58)	*p* value
Age (years)	48.6 ± 12.4	49.2 ± 13.1	0.798
Sex (Male/Female)	32/26	34/24	0.704
Type of poison			0.892
Pesticides	28	30	
Pharmaceuticals	18	16	
Industrial chemicals	8	7	
Other	4	5	
Time from poisoning to treatment (hours)	8.4 ± 3.2	8.6 ± 3.5	0.746
Baseline serum creatinine (mg/dL)	3.8 ± 1.2	3.9 ± 1.3	0.801
Baseline blood urea nitrogen (mg/dL)	42.5 ± 12.8	43.1 ± 13.4	0.804
Baseline 24-h urine output (mL)	385.0 ± 156.0	378.0 ± 162.0	0.812
APACHE II score	18.6 ± 4.8	19.1 ± 5.2	0.592
SOFA score	8.4 ± 2.6	8.6 ± 2.8	0.688

### Renal function parameters

**Figure 1:**
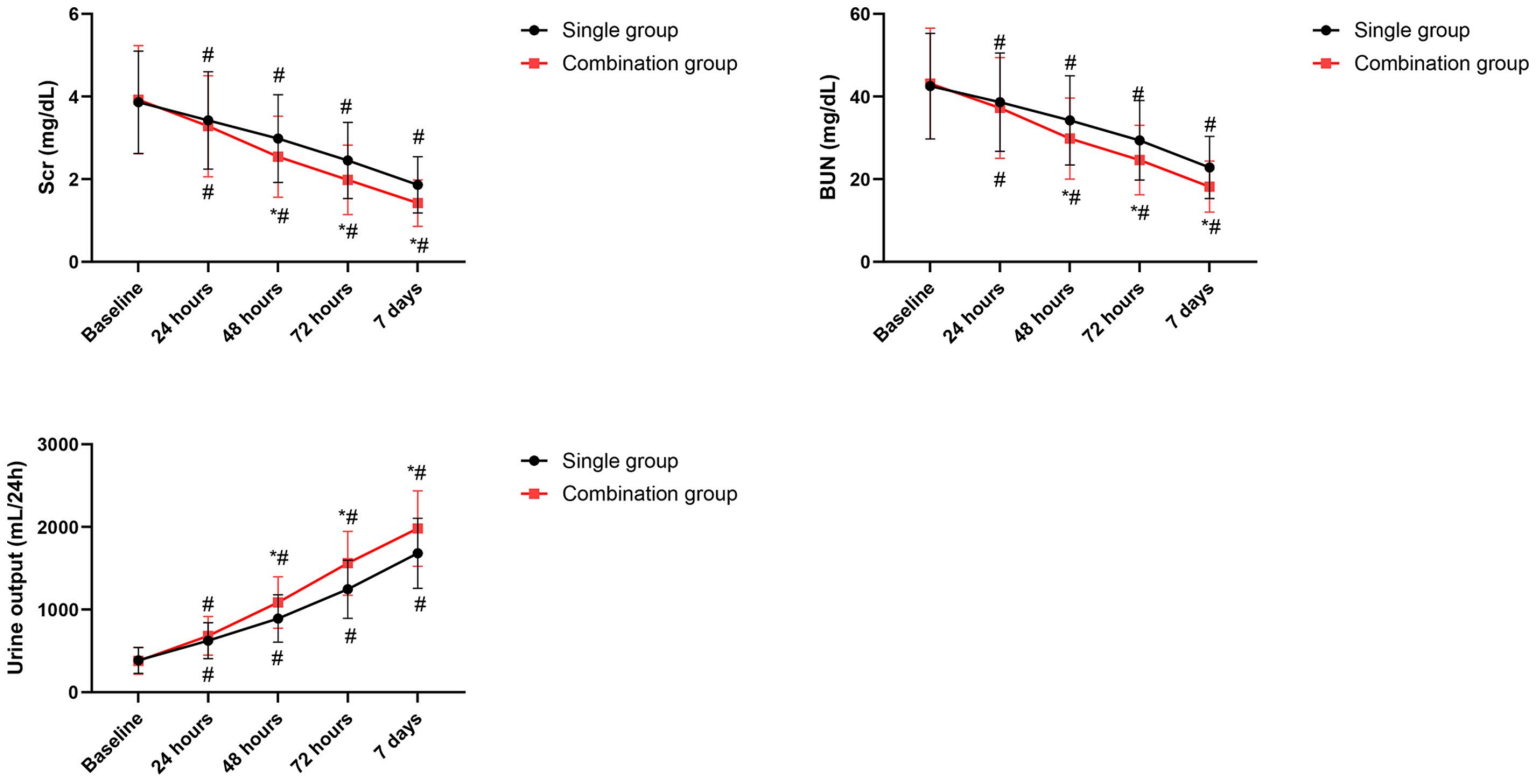
Changes in renal function parameters over time. Data are presented as mean ± SD. *p < 0.05 vs. single group. #*p* < 0.05 vs. baseline.

Both groups showed obvious reduction in Scr and BUN levels over time. However, the combination group exhibited significantly lower Scr and BUN levels at 48 h, 72 h, as well as 7 days post-treatment relative to the single group (*p* < 0.05 for all time points, Figure 1).

The urine output increased progressively in both groups, reflecting renal recovery. The combination group demonstrated significantly higher urine output at 48 h, 72 h, as well as 7 days post-treatment (*p* < 0.05 for all time points, Figure 1).

### Renal recovery

The proportion of patients achieving complete renal recovery (return to baseline Scr) at 28 days was significantly higher in the combination group than in the single group [48/58, 82.8% vs. 36/58, 62.1%; risk difference = 20.7% (95% CI: 5.6 to 35.8%), relative risk = 1.33 (95% CI: 1.06 to 1.68), number needed to treat = 5 (95% CI: 3 to 18), *p* = 0.014, Table 2].

**Table 2:** Proportion of patients achieving complete renal recovery (return to baseline Scr) at 28 days.

Groups	Cases	Complete renal recovery	RD (95% CI)	RR (95% CI)	NNT (95% CI)
Single group	58	36 (62.1%)	Reference	Reference	Reference
Combination group	58	48 (82.8%)	20.7% (5.6 to 35.8%)	1.33 (1.06 to 1.68)	5 (3 to 18)
*P* value		0.014			

### Immune function parameters

**Figure 2:**
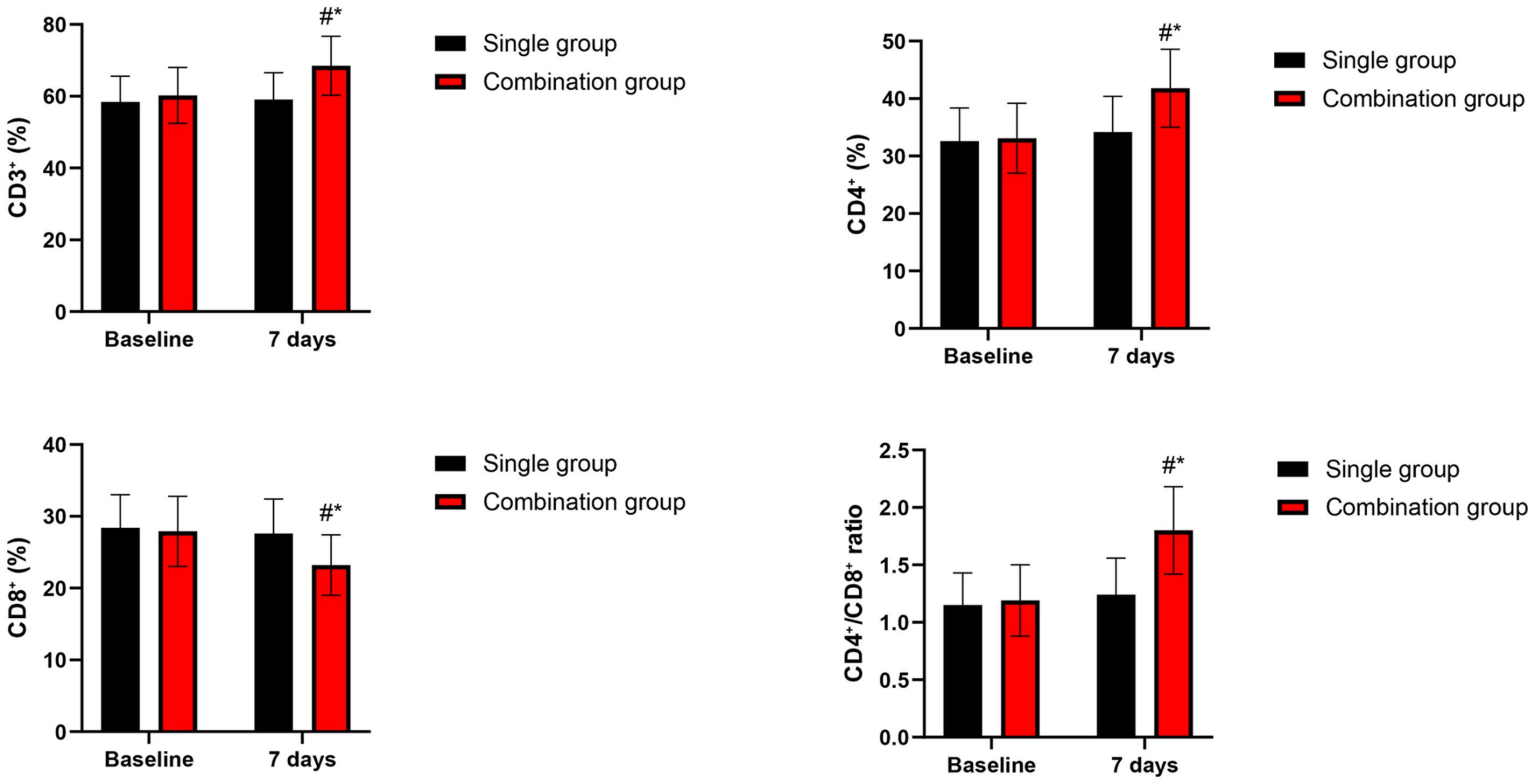
T lymphocyte subsets at baseline and 7 days post-treatment. Data are presented as mean ± SD. *p < 0.05 vs. single group. #*p* < 0.05 vs. baseline.

At baseline, no differences were seen between groups in any T lymphocyte subset parameters (*p* > 0.05). At 7 days post-treatment, the combination group demonstrated significantly higher CD3^+^, CD4^+^, and CD4^+^/CD8^+^ ratio, and significantly lower CD8^+^ relative to the single group (*p* < 0.05 for all comparisons). Within-group analyses showed that the combination group had significant improvements from baseline in all T lymphocyte parameters (*p* < 0.01), while the single group showed only modest, non-significant changes (Figure 2).

**Figure 3:**
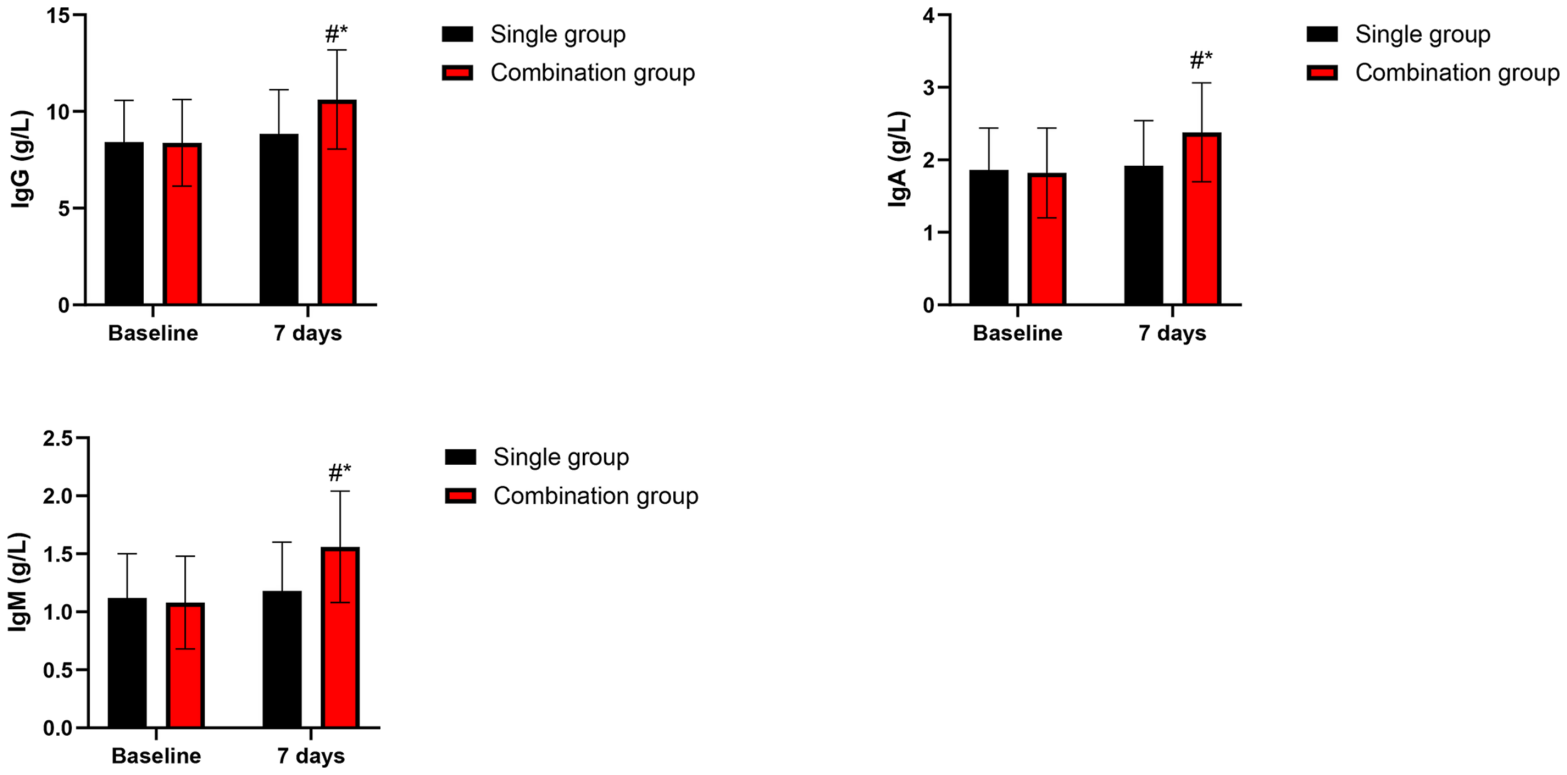
Serum immunoglobulins at baseline and 7 days post-treatment. Data are presented as mean ± SD. *p < 0.05 vs. single group. #*p* < 0.05 vs. baseline.

At baseline, immunoglobulin levels were comparable between groups. At 7 days post-treatment, the combination group presented higher levels of IgG, IgA, and IgM relative to the single group (*p* < 0.05 for all comparisons). The combination group presented significant increases from baseline in all immunoglobulin classes (*p* < 0.05), while the single group showed only modest, non-significant changes (Figure 3).

### Inflammatory markers

**Figure 4:**
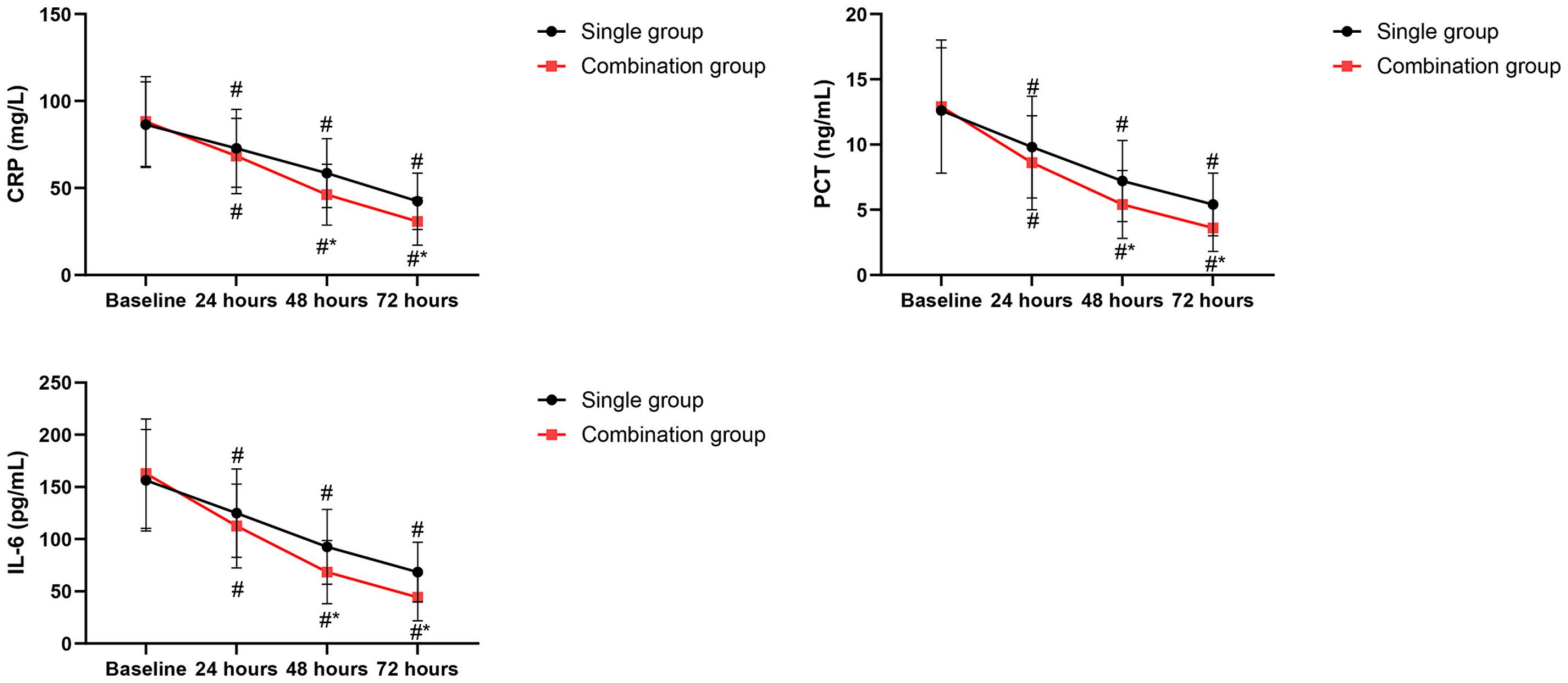
Changes in inflammatory markers over time. Data are presented as mean ± SD. *p < 0.05 vs. single group. #*p* < 0.05 vs. baseline.

Both groups showed obvious reduction in CRP, PCT as well as IL-6 levels over time. However, the combination group exhibited significantly lower CRP, PCT as well as IL-6 levels at 48 and 72 h post-treatment relative to the single group (*p* < 0.05, Figure 4).

### Disease severity scores

**Figure 5:**
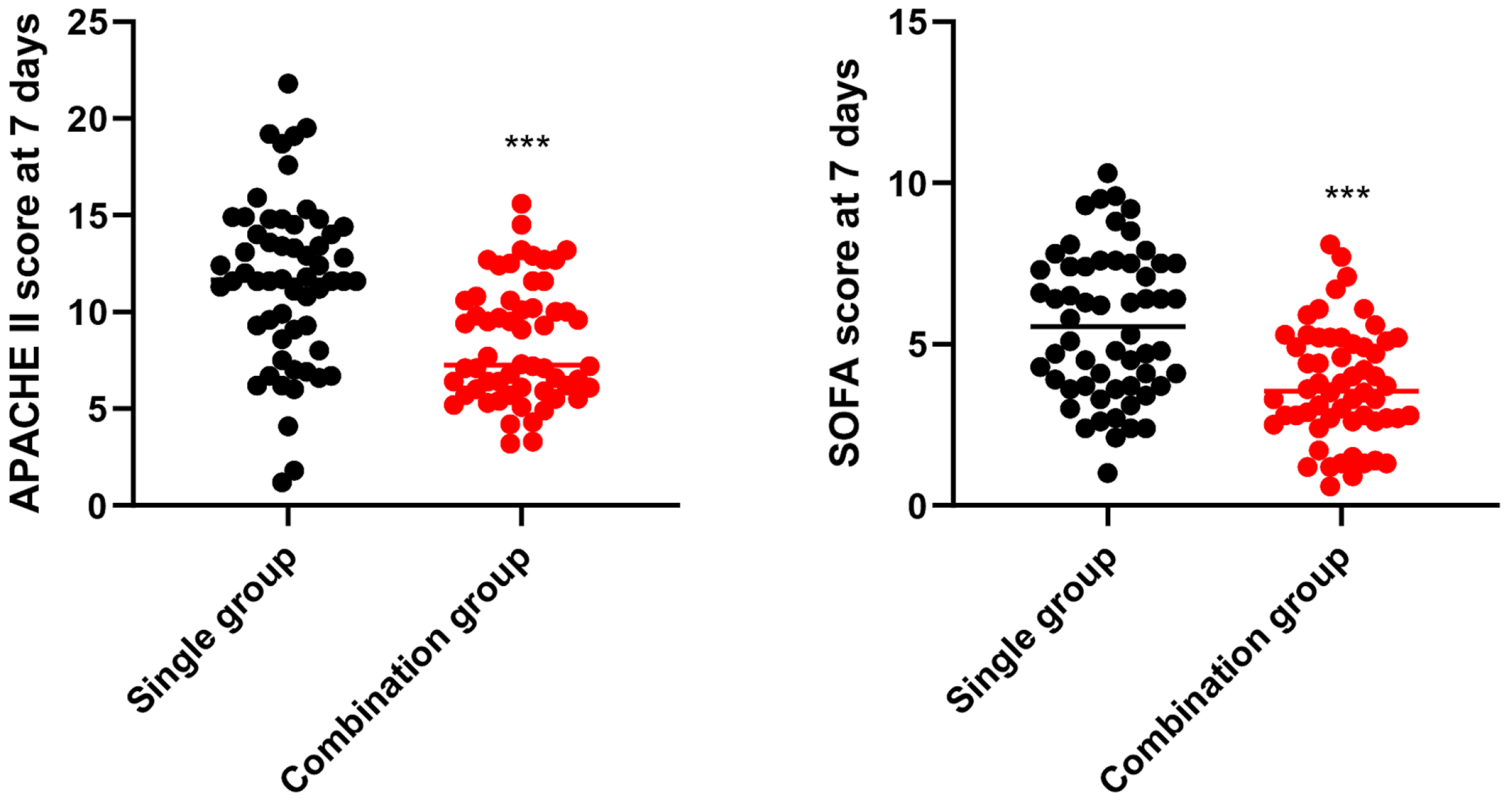
Disease severity scores at 7 days. ***p < 0.001 vs. single group.

At baseline, APACHE II and SOFA scores were comparable between groups. At 7 days, both groups presented significant improvement from baseline, but the combination group had significantly lower APACHE II scores (8.4 ± 3.0) and SOFA scores (3.8 ± 1.8) compared to the single group (11.6 ± 4.2 and 5.6 ± 2.2) (*p* < 0.001, Figure 5).

### Clinical outcomes

**Figure 6:**
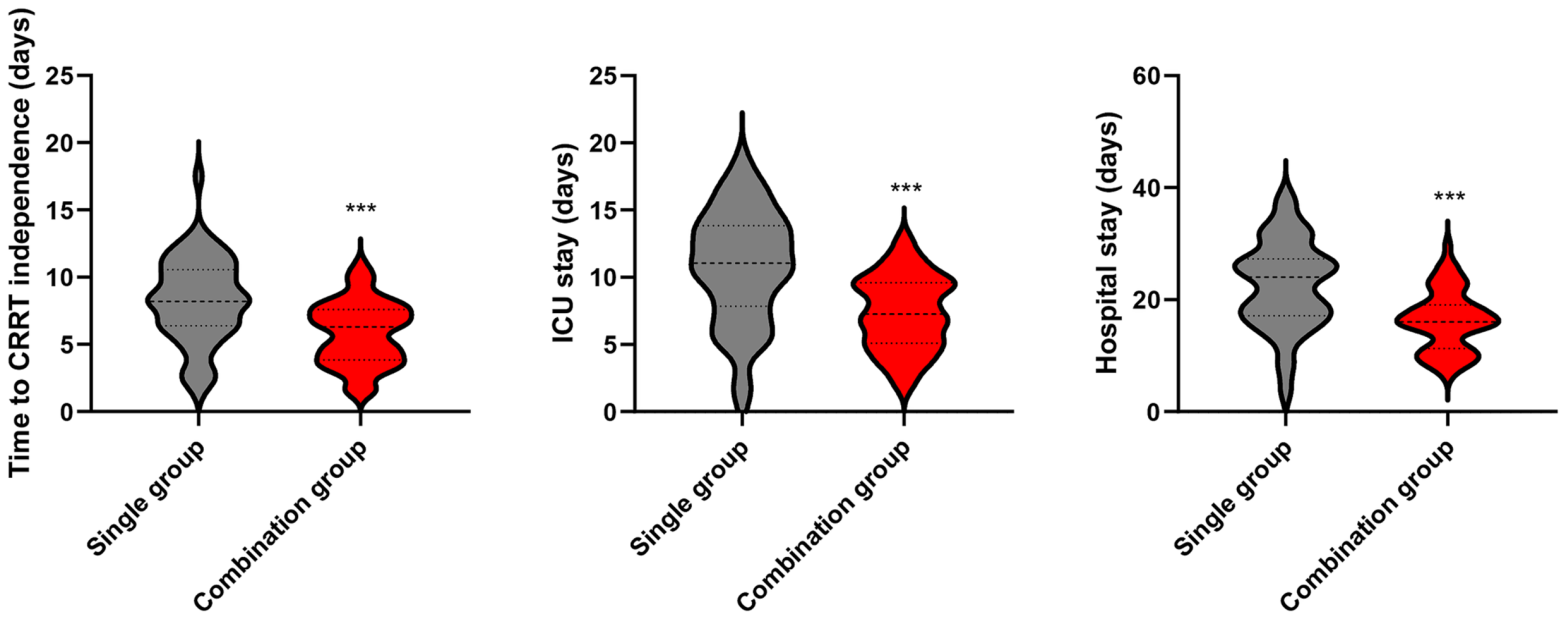
Time to CRRT independence, ICU stay, and hospital stay between the two groups. ***p < 0.001 vs. single group.

The combination group had significantly shorter time to CRRT independence, shorter ICU stay and total hospital stay (5.8 ± 2.4 days, 7.4 ± 2.8 days and 16.2 ± 5.4 days) relative to the single group (8.2 ± 3.1 days, 10.8 ± 4.2 days and 22.6 ± 7.8 days) (*p* < 0.001, Figure 6).

As shown in Table 3, the 28-day mortality rate was significantly lower in the combination group (6/58, 10.3%) compared to the single group (14/58, 24.1%) [risk difference = −13.8% (95% CI: −26.5% to −1.1%); relative risk = 0.43 (95% CI: 0.18 to 0.99); number needed to treat = 8 (95% CI: 4 to 91); *p* = 0.048]. The total incidence of adverse events presented lower in the combination group (10/58, 17.2%) compared to the single group (19/58, 32.8%) (*p* = 0.016). The most common adverse events were bleeding complications (single: 8 cases; combination: 4 cases), hypotension (single: 6 cases; combination: 4 cases), and catheter-related infections (single: 5 cases; combination: 2 cases).

**Table 3:** 28-day mortality rate and incidence of adverse reactions between the two groups.

Groups	Cases	28-day mortality rate	Risk difference (95% CI)	Relative risk (95% CI)	NNT (95% CI)	Bleeding	Hypotension	Catheter-related infections	Total incidence rate
Single group	58	14 (24.1%)	Reference	Reference	Reference	8	6	5	19 (32.8%)
Combination group	58	6 (10.3%)	−13.8% (−26.5% to −1.1%)	0.43 (0.18 to 0.99)	8 (4 to 91)	4	4	2	10 (17.2%)
*P* value		0.048							0.016

### Coagulation parameters and bleeding risk

To further evaluate the safety profile, we analyzed serial platelet counts and fibrinogen levels. As shown in Table 4, both groups experienced a mild decline in platelet count and fibrinogen at 48 h post-treatment, reflecting consumption coagulopathy associated with severe poisoning and extracorporeal circuit activation. However, at day 7, the combination group had significantly higher platelet counts (182 ± 34 × 10^9^/L vs. 156 ± 41 × 10^9^/L, *p* = 0.02) and fibrinogen levels (3.2 ± 0.6 g/L vs. 2.7 ± 0.7 g/L, *p* = 0.01) compared to the single group. Notably, despite undergoing three HP sessions, the combination group had fewer bleeding complications (4 cases vs. 8 cases in the single group). This paradoxical observation can be explained by the more frequent use of regional citrate anticoagulation (RCA) in the combination group (72.4% vs. 44.8% in the single group, *p* = 0.003), which provides effective circuit anticoagulation without systemic heparinization. Additionally, the combination group had significantly lower IL-6 levels (Figure 4), and reduced inflammatory activation likely attenuated platelet consumption and preserved fibrinogen. The shorter duration of CRRT exposure in the combination group (5.8 vs. 8.2 days) also contributed to reduced anticoagulant exposure time. Notably, a review of medical records confirmed that no patients in either group received plasma transfusion (fresh frozen plasma, platelets, or cryoprecipitate) during the study period, indicating that the observed differences in platelet count and fibrinogen reflect endogenous hemostatic recovery rather than exogenous supplementation. D-dimer and fibrin degradation products were not routinely measured, and circuit clotting events were not prospectively documented as a separate endpoint in either group; therefore, these data cannot be directly compared.

**Table 4:** Serial changes in platelet count and fibrinogen levels.

Time point	Platelet count (×10^9^/L)		Fibrinogen (g/L)	
	Single group (*n* = 58)	Combination group (*n* = 58)	Single group (*n* = 58)	Combination group (*n* = 58)
Baseline	198 ± 45	202 ± 48	3.5 ± 0.7	3.6 ± 0.8
48 h	142 ± 38	148 ± 40	2.6 ± 0.6	2.8 ± 0.6
Day 7	156 ± 41	182 ± 34*	2.7 ± 0.7	3.2 ± 0.6*

*p < 0.05 vs. single group.

### Hemodynamic and liver function parameters

To assess treatment safety and patient tolerance, we recorded mean arterial pressure (MAP), alanine aminotransferase (ALT), and aspartate aminotransferase (AST) at baseline, 48 h, and day 7. As shown in Table 5, MAP remained stable in both groups throughout the treatment period, with no significant differences between groups at any time point (*p* > 0.05 for all comparisons), indicating that neither CRRT alone nor HP + CRRT caused clinically significant hypotension beyond the underlying critical illness. Both groups showed elevated ALT and AST at baseline due to poisoning-related systemic inflammation and possible direct hepatotoxicity. At 48 h and day 7, liver enzymes decreased in both groups, reflecting clinical improvement. Notably, the combination group had significantly lower ALT and AST levels at day 7 compared to the single group (ALT: 38.5 ± 18.2 U/L vs. 52.3 ± 22.4 U/L, *p* = 0.01; AST: 42.1 ± 20.5 U/L vs. 58.6 ± 24.1 U/L, *p* = 0.01), suggesting that HP + CRRT may accelerate the resolution of liver injury, possibly by removing inflammatory cytokines and toxins. No patient in either group developed citrate accumulation-related hepatotoxicity (i.e., total Ca/iCa ratio >2.5 with concurrent liver dysfunction) when RCA was used.

**Table 5:** Serial changes in MAP, ALT, and AST.

Parameter	Time point	Single group (*n* = 58)	Combination group (*n* = 58)	*P* value
MAP (mmHg)	Baseline	72.4 ± 8.6	71.8 ± 9.2	0.72
	48 h	74.2 ± 7.8	75.1 ± 8.5	0.55
	Day 7	78.5 ± 6.4	79.2 ± 6.1	0.61
ALT (U/L)	Baseline	85.6 ± 32.4	88.2 ± 35.1	0.68
	48 h	62.3 ± 25.6	54.8 ± 22.4	0.09
	Day 7	52.3 ± 22.4	38.5 ± 18.2	0.01
AST (U/L)	Baseline	92.4 ± 38.2	95.1 ± 40.5	0.71
	48 h	68.5 ± 28.4	59.2 ± 24.6	0.07
	Day 7	58.6 ± 24.1	42.1 ± 20.5	0.01

## Discussion

This research investigated the impacts of HP + CRRT on renal function and immune function in patients with poisoning-induced ARF. Our findings demonstrate that HP + CRRT significantly improves renal function recovery, enhances immune function parameters, and reduces inflammatory markers compared to CRRT alone, leading to better clinical outcomes.

The rationale for combining HP with CRRT lies in their complementary mechanisms of action. CRRT provides continuous, slow removal of small molecular weight solutes and maintains acid–base and electrolyte balance through convection and diffusion (14). However, its efficacy in removing protein-bound or large molecular weight toxins—commonly encountered in severe poisoning—is limited (15). HP, by contrast, utilizes adsorbent materials such as neutral macroporous resins to directly bind and remove circulating toxins, including those with high protein-binding affinity (16). When used in series, the combined modality achieves enhanced clearance of both small and large molecular weight toxins, as well as protein-bound substances (17).

Our observation of improved renal function recovery in the HP + CRRT group is in accordance with previous reports. Cheng et al. reported that HP combined with CRRT effectively reduce the inflammatory response, and shorten the rescue and treatment time of patients with acute severe organophosphorus pesticide poisoning (12). Similarly, a study by Ma et al. (18) demonstrated that CRRT combined with HP can alleviate pulmonary function and prognosis of patients with severe acute pancreatitis.

A particularly noteworthy finding of our research is the beneficial effect of HP + CRRT on immune function. Severe poisoning is associated with profound immune dysregulation, characterized by imbalances in T lymphocyte subsets and excessive inflammatory responses (19). The adsorption capacity of hemoperfusion extends beyond exogenous toxins to include inflammatory mediators such as cytokines, which contribute to the systemic inflammatory response syndrome along with organ dysfunction (20). By removing both toxins and inflammatory mediators, HP + CRRT may help restore immune homeostasis. This immunomodulatory effect is supported by emerging evidence from studies using novel adsorption filters in sepsis-associated AKI (21). The oXiris filter, for example, has demonstrated superior capacity to remove pro-inflammatory cytokines and endotoxins while promoting renal protection (22).

The observed reduction in inflammatory markers (CRP, PCT, IL-6) in the HP + CRRT group provides further evidence of its immunomodulatory effects. Excessive inflammation is a key driver of both renal injury and distant organ dysfunction in poisoning (23). By attenuating the inflammatory response, HP + CRRT may break the vicious cycle of inflammation-induced injury and organ failure. This is consistent with findings from studies of polymyxin B hemoperfusion in endotoxic shock, where combined therapy resulted in faster decrease in endotoxin levels and trends toward improved outcomes (24). Furthermore, the faster decline in ALT and AST observed in the combination group aligns with previous evidence, including a network meta-analysis demonstrating comparable efficacy between charcoal hemoperfusion and other liver support devices in reducing mortality for non-paracetamol-poisoned patients with acute liver failure (25), as well as clinical studies showing that resin-based hemoperfusion effectively reduces ALT, AST, and total bilirubin in toxin-induced liver injury (26).

The efficacy of HP + CRRT may vary across different toxin types due to differences in physicochemical properties and adsorbability. For organophosphorus pesticides, a meta-analysis of 92 RCTs (6,899 patients) confirmed that HP combined with hemodialysis significantly improves rescue success and reduces complications (27). For paraquat, a 2026 systematic review concluded that early HP combined with CRRT demonstrates synergistic effects in improving survival (28). For diquat, HP combined with CRRT has been shown to effectively reduce serum diquat levels and improve renal outcomes (29). In the present study, due to the relatively small sample sizes of individual toxin subgroups (e.g., industrial chemicals, *n* = 15 total), we were unable to perform statistically meaningful subgroup analyses. Future large-scale studies with stratified randomization by toxin type are warranted to confirm the consistency of treatment effects across different poisoning agents.

Several factors may contribute to the superior outcomes observed with HP + CRRT. First, the timing of intervention is critical. Early initiation of combined therapy, as in our protocol, may prevent irreversible organ damage and modulate the inflammatory response before it becomes self-perpetuating. Second, the adsorption characteristics of the hemoperfusion cartridge are important. HA330 resin cartridges have been shown to effectively remove middle molecular weight substances, including cytokines, while maintaining good biocompatibility (30). Third, the continuous nature of CRRT ensures sustained metabolic control and prevents rebound phenomena that may occur with intermittent therapies. It should be noted that regional citrate anticoagulation was used more frequently in the combination group, and this may have contributed independently to the preservation of platelet count and fibrinogen; therefore, the observed improvement in hemostatic parameters cannot be attributed solely to hemoperfusion, although the accompanying reduction in inflammatory markers and fewer clinical bleeding events suggest a genuine benefit of the combined modality beyond the anticoagulation strategy.

### Limitations

Several limitations of this study should be acknowledged. First, this was a single-center trial with a relatively small sample size, which may limit generalizability; larger multi-center studies are needed to validate these findings. Second, the open-label design without blinding may have introduced performance and detection bias, particularly for subjective secondary outcomes such as adverse event assessment. Third, multiple secondary outcomes were analyzed without full adjustment for multiplicity, raising the possibility of type I error. Fourth, the heterogeneity of poisoning agents may have introduced variability in treatment response, and subgroup analyses by specific toxin were not feasible owing to limited sample size. Fifth, the optimal duration and frequency of hemoperfusion sessions remain undetermined; our protocol of three daily sessions was empirical. Sixth, the choice of a CRRT-only control group may limit applicability to centers where hemoperfusion is considered standard first-line therapy for severe poisoning, though the randomized design allowed quantification of the net benefit of adding HP to CRRT. Seventh, the relatively short 28-day follow-up limits insight into long-term renal and immune outcomes. Eighth, immune function assessment was limited to T-lymphocyte subsets and immunoglobulin levels; more detailed characterization of immune cell function would provide deeper mechanistic insights. Ninth, coagulation activation markers such as D-dimer and fibrin degradation products were not measured, and circuit clotting events were not systematically recorded. Tenth, anticoagulation was allocated based on clinical criteria rather than randomization, resulting in an imbalance in regional citrate anticoagulation use between groups and introducing residual confounding in the interpretation of coagulation-related outcomes. Finally, nafamostat mesylate, a short-half-life anticoagulant with potentially lower bleeding risk, was not used because it is not approved in our hospital formulary for continuous renal replacement therapy and its cost is prohibitive for routine use. Future studies with standardized anticoagulation protocols, systematic coagulation monitoring, and blinded outcome assessment are warranted to confirm these findings.

## Conclusion

In conclusion, hemoperfusion combined with continuous renal replacement therapy significantly improves renal function recovery and enhances immune function in patients with poisoning-induced acute renal failure compared to CRRT alone. The combined modality reduces inflammatory markers, shortens the duration of renal replacement therapy and ICU stay, and may improve survival. These findings suggest that HP + CRRT may be an effective therapeutic strategy for this critically ill patient population and provide a rationale for future larger, multi-center, blinded trials to confirm its potential role in poisoning management.

## Data Availability

The original contributions presented in the study are included in the article/Supplementary material, further inquiries can be directed to the corresponding author/s.
