# Supplementary figures and images for: Impacts of hemoperfusion combined with continuous renal replacement therapy on renal function and immune function in patients with acute renal failure caused by poisoning
Source: Front Med (Lausanne). 2026 May 20;13:1827840. doi: 10.3389/fmed.2026.1827840 (PMC13229827; doi:10.3389/fmed.2026.1827840)

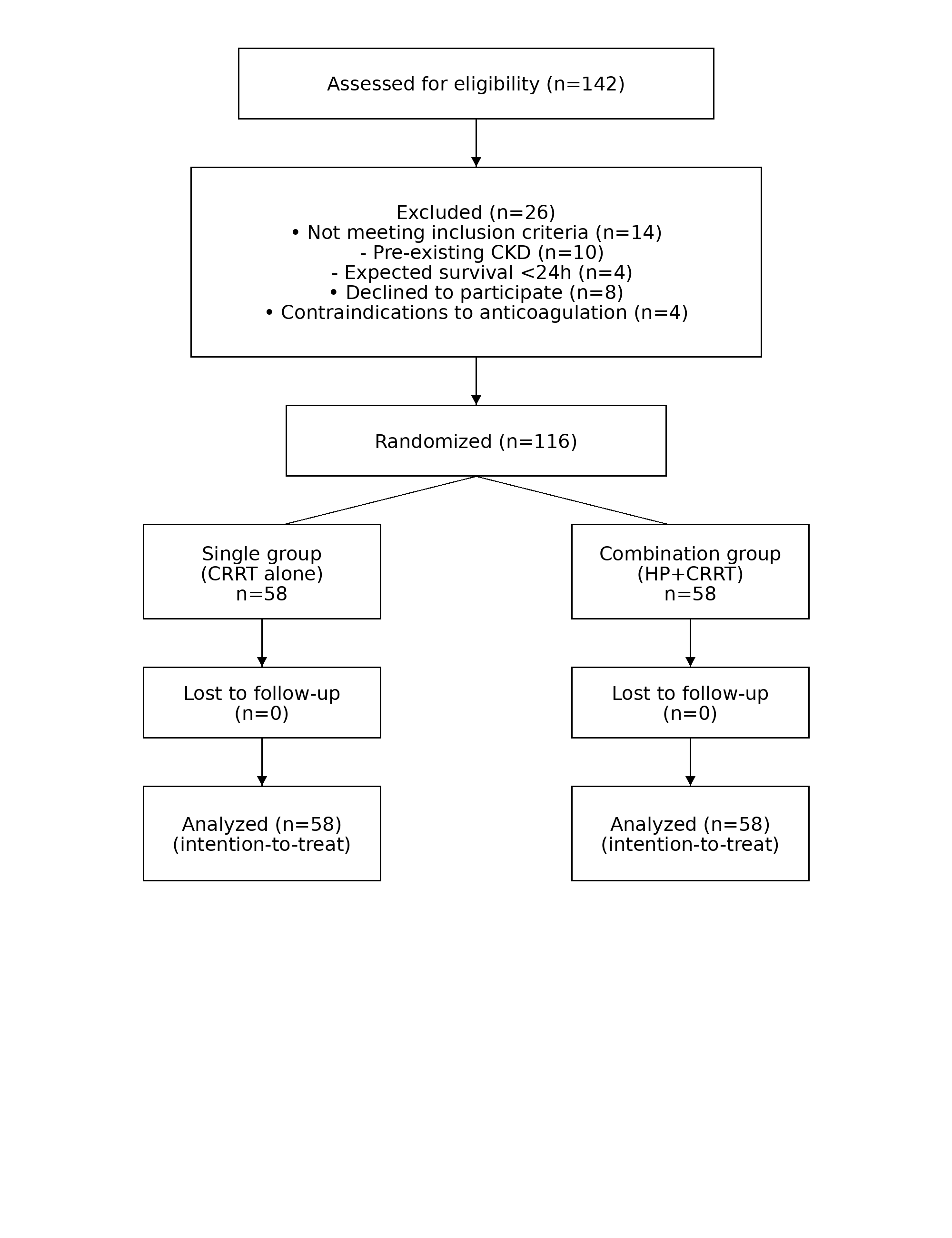

Supplement: Supplementary file 1 [file Image_1.tiff]
