# Supplementary material for: Impacts of hemoperfusion combined with continuous renal replacement therapy on renal function and immune function in patients with acute renal failure caused by poisoning
Source: Front Med (Lausanne). 2026 May 20;13:1827840. doi: 10.3389/fmed.2026.1827840 (PMC13229827; doi:10.3389/fmed.2026.1827840)
Supplement: Supplementary file 2 [file Table_1.docx]

**Detailed Nursing Management Protocol**

**Pre‑treatment nursing:** Before initiating CRRT or HP, nurses assessed patients‘ vital signs (heart rate, blood pressure, respiratory rate, oxygen saturation), fluid status (intake/output, edema, weight), and vascular access sites (redness, swelling, patency). Patients and families received detailed explanations about the purpose, procedure, and potential complications of CRRT and HP. Emergency equipment and medications were prepared at the bedside.

**Intra‑procedural monitoring:** Vital signs were monitored every 1‑2 hours. The CRRT circuit was closely observed for signs of clotting, air bubbles, or disconnections. For patients in the combination group, the hemoperfusion cartridge was monitored for saturation and pressure changes. Fluid balance was recorded every 2 hours, including input (dialysate, replacement fluid, medications) and output (ultrafiltrate, urine). Anticoagulation status was assessed via activated clotting time (ACT) or activated partial thromboplastin time (APTT) according to the protocol (see Anticoagulation protocol in Methods).

**Catheter care:** Strict aseptic technique was maintained during all manipulations of the central venous catheter. The insertion site was inspected daily for signs of infection (redness, swelling, exudate). Dressings were changed every 48 hours or immediately if soiled. Catheter patency was maintained by regular flushing with heparinized saline according to hospital protocol.

**Complication management:** For bleeding complications, pressure was applied to the insertion site, and anticoagulation was temporarily suspended under physician guidance. For hypotension, fluid resuscitation was initiated, and vasopressor support was administered as ordered. Signs of catheter‑related bloodstream infection (fever, chills, leukocytosis) prompted immediate blood culture collection and empirical antibiotic therapy.

**Nutritional and psychological support:** Nutritional status was assessed daily; enteral or parenteral nutrition was provided according to individual requirements. Psychological support was offered to address anxiety, fear, and depression. Family communication was maintained regularly.

**Post‑treatment care:** After treatment completion or at the end of each session, patients were assessed for hemodynamic stability, bleeding signs, and catheter site condition. The CRRT circuit was discontinued according to protocol, and the catheter was locked with heparin solution. Patients were monitored for at least 2 hours post‑treatment for delayed complications.

**Documentation:** All nursing assessments, interventions, and observations were documented in the electronic medical record system, including vital sign trends, fluid balance charts, complication occurrences, and responses to interventions.
